# Mangiferin Accelerates Glycolysis and Enhances Mitochondrial Bioenergetics

**DOI:** 10.3390/ijms19010201

**Published:** 2018-01-09

**Authors:** Zhongbo Liu, Pasha Apontes, Ekaterina V. Fomenko, Nan Chi, Victor L. Schuster, Irwin J. Kurland, Jeffrey E. Pessin, Yuling Chi

**Affiliations:** 1 Department of Medicine, Albert Einstein College of Medicine, Bronx, NY 10461, USA; liuzb727@gmail.com (Z.L.); p.apontes@gmail.com (P.A.); fomenko875ekaterina@gmail.com (E.V.F.); nanchi2013@yahoo.com (N.C.); victor.schuster@einstein.yu.edu (V.L.S.); Irwin.Kurland@einstein.yu.edu (I.J.K.); Jeffrey.Pessin@einstein.yu.edu (J.E.P.); 2 Department of Physiology and Biophysics, Albert Einstein College of Medicine, Bronx, NY 10461, USA; 3 Einstein Stable Isotope and Metabolomics Core, Albert Einstein College of Medicine, Bronx, NY 10461, USA; 4 Department of Molecular Pharmacology, Albert Einstein College of Medicine, Bronx, NY 10461, USA

**Keywords:** mangiferin, glucose, glycolysis, TCA cycle, mitochondrial bioenergetics, metabolomics, transcriptomics

## Abstract

One of the main causes of hyperglycemia is inefficient or impaired glucose utilization by skeletal muscle, which can be exacerbated by chronic high caloric intake. Previously, we identified a natural compound, mangiferin (MGF) that improved glucose utilization in high fat diet (HFD)-induced insulin resistant mice. To further identify the molecular mechanisms of MGF action on glucose metabolism, we conducted targeted metabolomics and transcriptomics studies of glycolyic and mitochondrial bioenergetics pathways in skeletal muscle. These data revealed that MGF increased glycolytic metabolites that were further augmented as glycolysis proceeded from the early to the late steps. Consistent with an MGF-stimulation of glycolytic flux there was a concomitant increase in the expression of enzymes catalyzing glycolysis. MGF also increased important metabolites in the tricarboxylic acid (TCA) cycle, such as α-ketoglutarate and fumarate. Interestingly however, there was a reduction in succinate, a metabolite that also feeds into the electron transport chain to produce energy. MGF increased succinate clearance by enhancing the expression and activity of succinate dehydrogenase, leading to increased ATP production. At the transcriptional level, MGF induced mRNAs of mitochondrial genes and their transcriptional factors. Together, these data suggest that MGF upregulates mitochondrial oxidative capacity that likely drives the acceleration of glycolysis flux.

## 1. Introduction

The hallmark of diabetes is hyperglycemia. One of the main causes of hyperglycemia is inefficient or impaired glucose utilization in skeletal muscle, which can be exacerbated by chronic high caloric intake. Following uptake, glucose metabolism takes place in three pathways, glycogen synthesis, the pentose phosphate shunt and glycolysis. The last one generates pyruvate, a key metabolic branching point at which carbohydrate metabolism either goes through the anaerobic pathway to form lactate or the aerobic pathway to generate energy in mitochondria. While formation of lactate is a relatively fast way for glucose metabolism, the oxidative metabolism in mitochondria produces much more energy and is a more effective pathway to utilize carbohydrate nutrients and eliminate energy storage in form of, for instance, fat.

The oxidative metabolism of carbohydrates in mitochondria is composed of two processes, the tricarboxylic acid (TCA) cycle and the electron transport chain (ETC). Pyruvate enters the TCA cycle mainly via two possible pathways, pyruvate decarboxylation by pyruvate dehydrogenase (PDH) to form acetyl-CoA, whose subsequent metabolites feed into the ETC for mitochondrial respiration to produce energy, or carboxylation by pyruvate carboxylase to form oxaloacetate, which participates in TCA cycle anaplerosis [1,2]. The majority of pyruvate enters the TCA cycle via the irreversible pyruvate decarboxylation by PDH [2]. In addition, there are two other irreversible reactions in the TCA cycle, citrate synthase (CS) catalyzed formation of citrate from acetyl-CoA and α-ketoglutarate dehydrogenase (α-KGDH) catalyzed formation of succinyl-CoA from α-KG. Based on the reversibility of catabolic reactions, the TCA cycle is thought to operate in two sequential segments, the first being from acetyl-CoA until α-KG and the second being from α-KG to oxaloacetate [3].

Impaired glycolysis and mitochondrial bioenergetics have been reported under pathological conditions such as obesity and diabetes [4]. Previous studies have identified several enzymatic steps that are dysregulated in diabetes in vivo or under hyperglycemic conditions in vitro. In particular, phosphofructose kinase (PFK) and glyceraldehyde 3-phosphate dehydrogenase (GAPDH) are down-regulated in terms of both expression and activity [5,6,7,8,9,10,11,12]. Significantly reduced resting metabolic flux through the TCA cycle and oxidative phosphorylation were reported in skeletal muscle of patients with type 2 diabetes and insulin resistant offspring of type 2 diabetic patients [13,14,15]. Reduced metabolites in the TCA cycle were also reported in high fat diet (HFD)-fed mice [2,16]. In parallel with reduced metabolic flux, mitochondrial DNA (mtDNA) and the transcription of mitochondrial genes are also suppressed in skeletal muscle in insulin-resistant individuals [17,18]. It has been proposed that correction of impaired catalytic steps in glycolysis and mitochondrial bioenergetics could mitigate hyperglycemia. Thus, there has been great interest in identifying mechanisms that increase glycolysis and mitochondrial bioenergetics, and based on such mechanisms identifying nutraceuticals/pharmaceuticals that can improve glucose homeostasis.

MGF is a C-glucosyl xanthone (C2-β-d-glucopyranosyl-1,3,6,7-tetrahydroxyxanthone) that is a predominant constituent in extracts of the mango plant *Mangifera indica* L. Recently, we reported that MGF can enhance insulin sensitivity and mitigate glucose intolerance in HFD-induced insulin resistant mice [19]. In addition, MGF enhanced glucose oxidation both in cultured C2C12 myotubes and, particularly interestingly, in skeletal muscle isolated from obese and insulin resistant mice induced by HFD feeding. We have also demonstrated that MGF activates PDH, which presumably directs glucose metabolism to the mitochondrial oxidative processes. However, detailed information on how MGF affects each step of glycolysis and mitochondrial oxidative metabolism remains unknown. In this study, we investigated the molecular mechanisms responsible for the metabolic beneficial effects of MGF by using targeted metabolomics and transcriptomics.

## 2. Results

### 2.1. Profiling of Metabolites in Glucose Metabolism

In our previous study, we found that MGF orally administered to mice on HFD was able to reduce plasma glucose and insulin levels [19]. We also found that MGF enhanced carbohydrate oxidation in skeletal muscle of mice fed either chow or HFD [19]. Thus, we proposed that the MGF’s abilities of sensitizing insulin signaling and lowering glucose is, at least partially, due to MGF enhanced oxidative utilization of glucose in skeletal muscle. The processes of oxidative utilization of carbohydrates include glycolysis, and subsequent entry into the TCA cycle and the ETC. To understand how MGF affected these metabolic processes, we isolated quadriceps skeletal muscle from mice maintained on a normal low fat or HFD and were treated with or without MGF for 16 weeks, as previous reported [19] and analyzed metabolites in those skeletal muscle tissues.

Quantitative targeted metabolomics of several metabolic intermediates are listed in Table 1. Among all metabolites listed in Table 1, HFD significantly increased one metabolite, glucose-6-phosphate. Conversely, it significantly reduced several other metabolites in the glycolysis and the TCA cycle including phosphoenolpyruvate (PEP), pyruvate, α-KG, succinate, fumarate and aspartate. Overall MGF significantly affected more metabolites in HFD-fed mice than in chow fed mice.

### 2.2. MGF Accelerates Glycolysis

#### 2.2.1. MGF Increases Glucose Metabolism via Glycolysis

**Figure 1:**
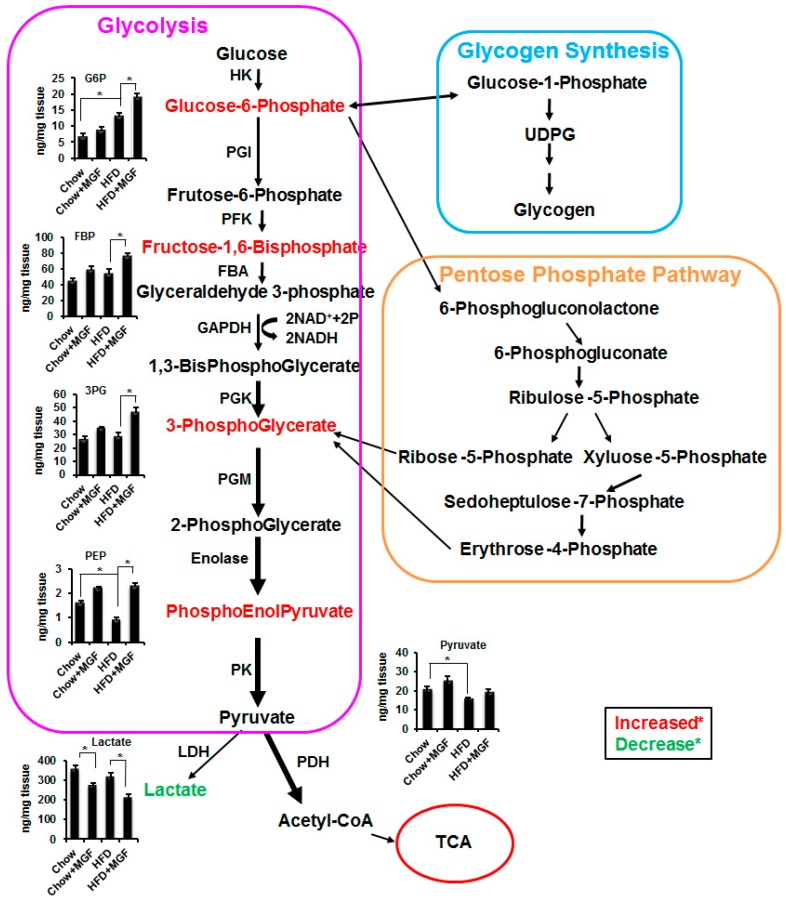
MGF accelerates glycolysis. Glucose metabolic pathways include glycogen synthesis, pentose phosphate shunt and glycolysis. Arrows in the glycolysis pathway indicate mangiferin directed metabolic flux from one product to the next. Arrows in other pathways mean change of one metabolic product to the other. Significantly altered metabolites are presented in bar graphs. Values are average ± SEM (*n* = 6). * *p* < 0.05. HK, Hexose kinase; PGI, Phosphoglucose isomerase; PFK, Phosphofructokinase; FBA, Fructose-1,6-bisphosphate aldolase; GAPDH, Glyceraldehyde 3-phosphate dehydrogenase; PGK, Phosphoglycerate kinase; PGM, Phosphoglycerate mutase; PK, Pyruvate kinase; PEP, phosphoenolpyruvae; LDH, lactate dehydrogenase; PDH, pyruvate dehydrogenase; UDPG, urindine diphosphate glucose.

To more clearly demonstrate the effects of MGF in the context of metabolic pathways, we present significantly altered metabolites in bar graphs in the pathways (Figure 1). The first metabolite of glucose is glucose-6-phosphate, which can be metabolized by three pathways, glycogen synthesis, the pentose phosphate shunt, and glycolysis. Urindine diphosphate glucose (UDPG) is the product of glucose-6-phosphate and precursor of glycogen. MGF did not significantly affect UDPG in either chow or HFD-fed mice (Table 1), suggesting that MGF probably did not affect glycogen synthesis pathway (Figure 1). As to the pentose phosphate shunt, MGF did not significantly affect 6-phosphogluconate (an important intermediate metabolite), nor ribose-5-phosphate (an important terminal metabolite) (Table 1), suggesting that MGF probably did not exert any significant effects on this pathway (Figure 1). In contrast, MGF increased glucose 6-phosphate, F-1,6-bisphosphate, 3-phosphoglycerate and PEP in chow and/or HFD-fed mice. The magnitude of these differences became greater, i.e., the ratios (MGF/Ctl) increased, as glycolysis proceeded from the early steps to the late steps, indicating that MGF accelerated metabolic flux via the glycolysis pathway. The effects are more significant and profound in HFD-fed mice than in chow mice (Table 1, Figure 1).

#### 2.2.2. MGF Induces Some Enzymes in Glycolysis

**Figure 2:**
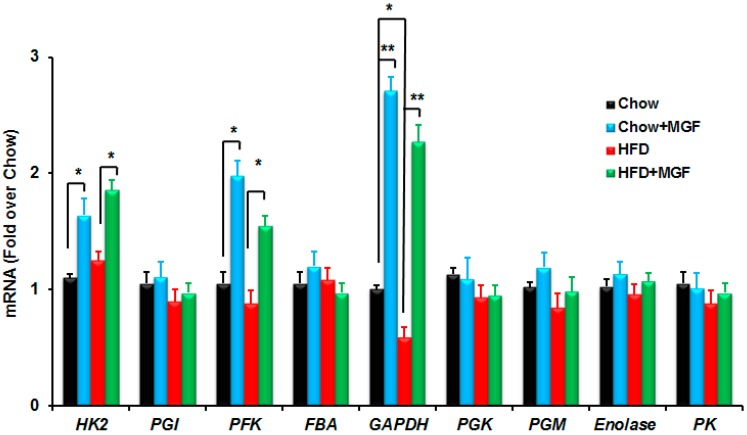
MGF induces mRNAs of some enzymes participating in glycolysis. Profiles of mRNAs of glycolytic enzymes in quadriceps from mice treated with chow or HFD ± MGF for 16 weeks. Values are average ± SEM (*n* = 6/group). * *p* < 0.05, ** *p* < 0.01.

To further understand how MGF might alter the metabolites in glycolysis, we profiled mRNAs of those enzymes in muscle tissues from those chow or HFD-fed mice treated with or without MGF. HFD significantly reduced the mRNA of *GAPDH*, whereas HFD had no significant effect on the other glycolytic enzyme mRNAs examined (Figure 2). However, MGF treatment induced the mRNAs of three enzymes, *HK*, *PFK*, and *GAPDH*, in both chow and HFD-fed mice. Among these enzymes *GAPDH* is the one on which MGF exerted the greatest effect (Figure 2).

### 2.3. MGF Enhances Mitochondrial Bioenergetics

#### 2.3.1. MGF Enhances Metabolism in the TCA Cycle

Accelerated glycolysis by itself would lead to an increase in the end product, pyruvate. However, there was no significant increase in pyruvate levels in the MGF treated mice, suggesting a more rapid clearance of pyruvate. Pyruvate can be catabolized to several products via different pathways. Pyruvate can be converted to lactate by lactate dehydrogenase (LDH) via the anaerobic pathway or enter the TCA cycle to be metabolized via the aerobic pathway. Figure 1 shows that there is significantly less lactate produced in MGF treated mice, suggesting that the MGF-increased clearance of pyruvate was not via the anaerobic pathway in the cytosol, but rather via the TCA cycle occurring in mitochondria.

**Figure 3:**
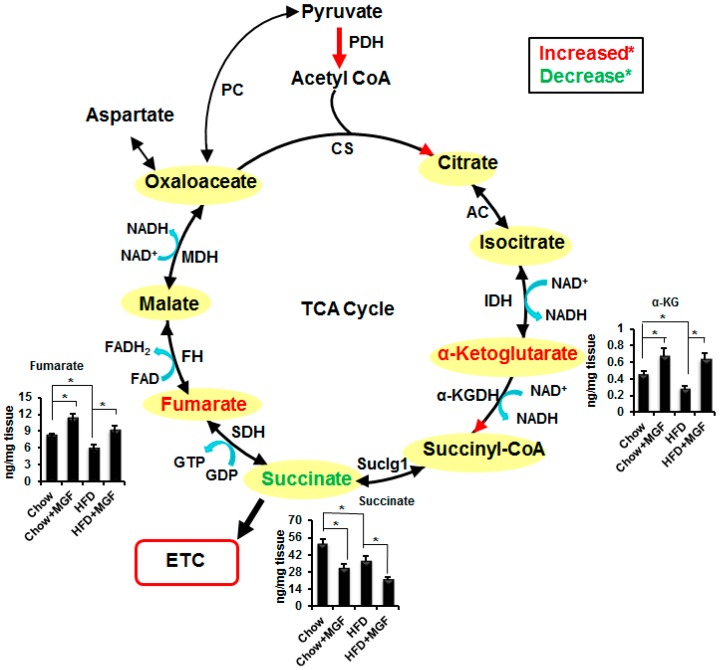
MGF modulates metabolite profiles in the TCA cycle. One-end arrows mean unidirectional and two-end arrows mean bidirectional. Significantly altered metabolites are presented in bar graphs. Values are average ± SEM (*n* = 6). * *p* < 0.05. PC, pyruvate carboxylase; PDH, pyruvate dehydrogenase; CS, citrate synthase; AC, Aconitase; IDH, isocitrate dehydrogenase; α-KGDH, α-ketoglutarate dehydrogenase; Suclg, succinyl coenzyme A synthetase; SDH, succinate dehydrogenase; FH, fumarase; MDH, malate dehydrogenase; α-KG, α-ketoglutarate.

In mitochondria, the majority of pyruvate is catabolized to acetyl CoA by PDH [2]. Acetyl CoA is then further metabolized to α-KG, which is a marker of the TCA flux during the first segment. We have previously reported that MGF activates PDH [19], and therefore we hypothesized that MGF accelerated the first segment of the TCA cycle. Indeed, MGF significantly increased α-KG, by about 150% in chow mice and by 220% in HFD muscle (Table 1, Figure 3). Interestingly, MGF elevated α-KG did not result in any increase in the subsequent metabolite of α-KG, succinate. Rather, MGF significantly reduced succinate by about 40% in chow and HFD muscles. On the other hand, MGF significantly increased fumarate formed from succinate by SDH by 138% in chow mice and by 151% in HFD muscle (Table 1, Figure 3). Fumarate is in rapid exchange with other four-carbon intermediates, malate and oxaloacetate, which can be reversibly converted to aspartate. MGF increased all these metabolites in the second segment of the TCA cycle, albeit some increases were insignificant.

#### 2.3.2. MGF Enhances SDH Activity and Overall Energy Production in Mitochondria

**Figure 4:**
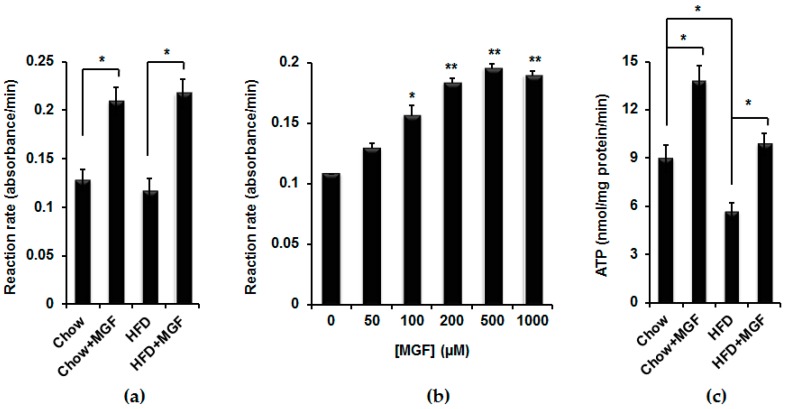
MGF enhances SDH activity and increases ATP production. Mitochondria were isolated from quadriceps of mice treated with chow or HFD ± MGF for 16 weeks (*n* = 6/group) (**a**) or C2C12 myotubes treated with various concentrations of MGF for 4 h (**b**). In vitro experiment presented in (**b**) was repeated for three times. The comparisons are between the rate at each MGF concentration with the rate when [MGF] = 0; (**c**) ATP production in freshly isolated mitochondria from quadriceps of mice treated with chow or HFD ± MGF for 16 weeks (*n* = 6/group). Values are average ± SEM. * *p* < 0.05, ** *p* < 0.01.

Succinate is an intermediate between α-KG and fumarate that were both increased, suggesting that the decrease in succinate levels was due to enhanced succinate consumption. MGF significantly increased the activity of SDH in mitochondrial isolated from quadriceps by 63% and 86% in mice fed with chow and HFD, respectively (Figure 4a). To confirm these in vivo effects, we treated C2C12 myotubes with various concentrations of MGF for about 4 h and isolated mitochondria from those cells and measured SDH activity. MGF also significantly increased SDH activity in those cells in a dose-dependent manner (Figure 4b).

Among the metabolites in the TCA cycle, succinate is the only one that enters the ETC to participate in a chain of oxidative reactions to ultimately produce energy, ATP. SDH is also the mitochondrial complex II in the ETC. SDH activity is a marker of mitochondrial oxidative capacity and positively related to energy production [20]. Thus, we measured ATP production in freshly isolated mitochondria from snap frozen quadriceps. HFD significantly reduced ATP production (Figure 4c). MGF significantly increased ATP production by about 150% in chow mice. Moreover, MGF reversed the reduction in ATP in HFD mice and increased the level of ATP slightly higher than that in chow control mice.

#### 2.3.3. MGF Induces Mitochondrial Biogenesis

**Figure 5:**
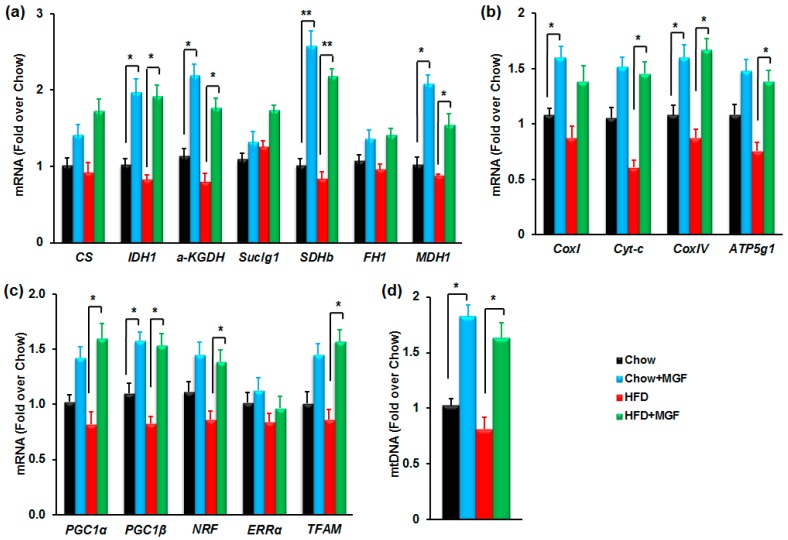
MGF induces mitochondrial biogenesis. (**a**–**c**) Profiles of mRNAs of mitochondrial genes (**a**,**b**) and transcriptional factors (**c**) in quadriceps of mice treated with chow or HFD ± MGF for 16 weeks (*n* = 6/group); (**d**) Relative mitochondria DNA contents in quadriceps of mice treated with chow or HFD ± MGF for 16 weeks. Values are average ± SEM (*n* = 6). * *p* < 0.05, ** *p* < 0.01.

To investigate whether MGF has any effects on the expressions of mitochondrial genes, we profiled mRNAs of all enzymes in the TCA cycle and the ETC from the quadriceps skeletal muscle. HFD feeding caused reduction in most of mitochondrial genes, although most of these changes did not reach statistical significance (Figure 5a,b). On the other hand, MGF significantly induced several genes in the TCA cycle and the ETC in chow and/or HFD-fed mice (Figure 5a,b). These genes included *IDH*, *αKGDH*, *SDH*, *CoxI*, *Cyt-c*, *COXIV* and *ATP5g1*. The greatest effects occur in *SDH*, which could be a factor contributing MGF enhanced catabolism of succinate to fumarate.

Mitochondrial genes are tightly regulated by several transcription factors including transcriptional factor A (*TFAM*), nuclear respiratory factor 1 (*NRF1*) and estrogen-related receptor α (*ERRα*), and the co-factors of these transcriptional factors, peroxisome proliferator-activated receptor-γ coactivator (*PGC)-1α* and *PGC-1β* [21,22,23,24,25,26]. MGF significantly increased all of those transcriptional factors, except *ERRα*, in chow or HFD-fed mice (Figure 5c), indicating that MGF upregulates those enzymes in the TCA cycle and the ETC at the transcriptional level. *PGC-1α* is positively correlated to mtDNA. Therefore, we measured mtDNA contents in mitochondria isolated from those muscle tissues. MGF almost doubled mtDNA in both chow and HFD-fed mice (Figure 5d). Together, these results indicate that MGF induces mitochondrial biogenesis.

## 3. Discussion

Obese, insulin resistant and diabetic patients display impaired glycolysis and dysfunctional mitochondrial oxidation [4,13,14,15,17,18], which are major contributors to impaired glucose disposal/utilization and hence to hyperglycemia. Pharmaceutical and/or nutraceutical agents that can improve glycolysis and mitochondrial oxidative capacity have the potential for therapeutic value in treating diet induced obesity and insulin resistance. Using a HFD mouse model of obesity and insulin resistance, we previously identified a natural compound, MGF, that was able to correct HFD induced glucose intolerance and insulin resistance by enhancing glucose utilization [19]. Using metabolite and transcriptomic profiling we have found that MGF gradually increased metabolic flux in glycolysis, which was facilitated by MGF induced enzymes in glycolysis. In mitochondria, MGF directs pyruvate metabolism in sequence from the first segment to the second. In such manner MGF enhances oxidative metabolism, resulting in increased production of energy, ATP. MGF does so by upregulating transcription of mitochondrial genes and enhancing the activity of SDH.

Glucose-6-phosphate is the first metabolite in glucose metabolism. HFD feeding almost doubled glucose-6-phosphate probably due to HFD blunted metabolism at the subsequent metabolic steps, as there was gradually augmented reduction in metabolites from the early steps to the later. Near the end of glycolysis, HFD caused significant reduction in PEP and pyruvate (Table 1, Figure 1). On the contrary, MGF gradually and significantly increased metabolites and the increases were augmented from the early steps to the later (Table 1, Figure 1). These results indicate that MGF is able to counteract the inhibitory effects of HFD on glycolysis.

The conversion of glucose to glucose-6-phosphate is catalyzed by HK. Previously Sellamuthu et al. [27] showed that MGF increased HK activity in liver of streptozotocin induced diabetic rats. Here we show that MGF induces HK expression in muscle of both chow and HFD-fed mice (Figure 2). In glycolysis, glucose-6-phosphate is subsequently converted to fructose-1,6-bisphosphate by PFK. While the effects of HFD on the activity of PFK is uncertain [28,29,30], diabetes reduces the expression and activity of PFK [31]. Deficiency in PFK is associated with impaired insulin secretion and insulin resistance in human [5,6,8,32]. MGF induces PFK (Figure 2) and therefore has the possible capability of mitigating PFK deficiency in diabetes. MGF also significantly induced the mRNA of *GAPDH*, which is a key enzyme in the glycolytic conversion of glucose to pyruvate and has been considered to play a regulatory role in setting the glycolytic rate [33,34]. The reports on the effects of HFD feeding on expression of *GAPDH* are inconsistent probably due to tissue specificity [35,36,37]. Here we show that HFD significantly reduced *GAPDH* mRNA in skeletal muscle (Figure 2). More importantly, MGF treatment caused more than 2-fold induction in mRNA of *GAPDH* in both chow and HFD-fed mice (Figure 2).

The end product of glycolysis is pyruvate, which can be converted to lactate via the anaerobic process or enter mitochondria to be metabolized by the aerobic process. While HFD did not cause significant change in lactate, it significantly reduced several key metabolites in the TCA cycle (Table 1, Figure 3), which was also reported by others [2,16]. MGF modulated those metabolites in a biologically important manner. Pyruvate is converted from PEP by PK. MGF increased PEP, but did not suppress PK. One would expect that there could be similar magnitude of increase in pyruvate in MGF treated mice. However, MGF only caused mild and insignificant increase in pyruvate, suggesting that MGF probably enhanced pyruvate consumption, either by increasing lactate production or by enhancing oxidative metabolic flux in the TCA cycle. MGF significantly reduced lactate in muscle tissues of both chow and HFD-fed mice (Table 1, Figure 1), which recapitulates the effects of MGF on lactate in cultured C2C12 myotubes shown in our previous study [19]. We further demonstrated that MGF reduced lactate production not by inhibiting pyruvate carboxylase (lactate dehydrogenase), but by enhancing PDH activity, shuttling pyruvate to mitochondria. In mitochondria PDH catalyzes the irreversible conversion of pyruvate to acetyl-CoA, which will be irreversibly converted to citrate by citrate synthase (CS). These unidirectional reactions make commitment to the flow of the TCA cycle to produce the end product of the first segment of the TCA cycle, α-KG. The fact that MGF increased α-KG, as shown in this study (Table 1, Figure 3), confirms that MGF enhances PDH and accelerates the metabolism in the first segment of the TCA cycle. This enhancement is also supported by MGF increased CS and, more significantly, IDH (Figure 5a).

Subsequently, α-KG is irreversibly converted to succinyl-CoA by α-KGDH. MGF significantly increased α-KGDH, presumably increasing succinyl-CoA, the starting point of the second segment of the TCA cycle, leading to the production of oxaloacetate. All of the reactions in the second segment of the TCA cycle are reversible and some of the metabolites are in rapid exchange with each other. MGF increased most metabolites in the second segment of the TCA cycle, except succinate. Succinate is a unique metabolite. It is the only metabolite in the TCA cycle that also feeds in the ETC and participates in oxidative phosphorylation. Accumulation of succinate is a marker of anaerobic metabolism [3]. MGF significantly reduced succinate, which supports the idea that MGF enhances oxidative metabolism.

MGF eliminates succinate probably by enhancing SDH, as MGF upregulates SDH at both the expression and the activity levels (Figure 4a,b and Figure 5a). SDH is the only enzyme that is a component of both the TCA cycle and the ETC. In the ETC, it is named complex II. SDH catalyzes the oxidation of succinate to fumarate and uses the electrons derived from this oxidation to catalyze the reduction of ubiquinione to ubiquinol. These electrons are passed to complex III and then complex IV, thereby contributing to the establishment of the electrochemical gradient across the mitochondria inner membrane for ATP synthesis [38]. Among all mitochondrial complexes, complex II loses activity most rapidly after death and on inappropriate storage, and therefore its activity is considered as a marker of the integrity of the tissue and the reliability of respiratory chain enzymology [20]. Mutation of SDH gene results in mitochondrial respiratory chain deficiency [39]. Reduced SDH expression and activity were reported in obese and diabetic patients [18]. Here we show that MGF upregulates SHD at both expression and activity levels in quadriceps of not only wild type mice but also, importantly, in obese and insulin resistant mice (Figure 4a,b and Figure 5a). MGF was also shown to enhance SDH activity in soleus and tibialis of obese rats, and to increase SDH activity in heart of experimentally induced myocardial infarcted rats [40,41].

The effects of MGF on mitochondria are not limited to SDH. In addition to SDH, MGF was shown to increase the activities of other important enzymes in the TCA cycle and the ETC, including α-KGDH, MDH and cytochrome c [41]. At the expression level, here we found that MGF induced mRNAs of most enzymes in the TCA cycle and the ETC (Figure 5a,b). Furthermore, MGF induced several important mitochondrial transcriptional factors and their co-factors including TFAM, NRF1, PGC-1α and PGC-1β (Figure 5c), as well as increasing mitochondrial DNA content (Figure 5d). Taking together, these results indicate that MGF enhanced overall mitochondrial oxidative capacity, which probably drives the accelerated metabolic flux in glycolysis (Figure 1).

In addition to accelerating glycolysis, MGF has been shown to reduce glucose 6-phosphate and fructose bisphosphatase and therefore inhibit gluconeogenesis [27]. These effects, together, suggest that MGF could be an effective anti-hyperglycemia and anti-diabetes agent. Furthermore, we and others have shown that MGF has anti-hyperlipidemia effects in rodents [19,42,43,44,45,46,47], and impressively in humans [48]. As a natural compound, MGF has been shown to uniquely possess multi-beneficial effects in mitigating metabolic disorders. The drawback is that it has very low bioavailability (1.2%) [49,50,51] and therefore the dose required for MGF to exert significant effects is high. Nevertheless, the structural features of MGF allow chemical modifications and MGF has the potential to be developed into an effective nutraceutical/pharmaceutical for the treatment of complex metabolic syndrome.

## 4. Materials and Methods

Chemical reagents were purchased from Sigma-Aldrich (St. Louis, MO, USA) unless specified.

### 4.1. Cell Culture

C2C12 myoblast cells were maintained in DMEM High Glucose (Hyclone, Logan, UT, USA), supplemented with 1% penicillin and streptomycin (GIBCO, Carlsbad, CA, USA) and 10% FBS. C2C12 cells were differentiated into myotubes in the above DMEM medium containing 2% horse serum (replacing 10% FBS) for 4–6 days after cells reached confluence.

### 4.2. Animal Experiments

Wild type C57BL6/J mice at age of 5–6 weeks were purchased from Jackson laboratory (Bar Harbor, ME, USA). All studies were approved by and performed in compliance with the guidelines of the Einstein Institutional Animal Care and Use Committee. Chow diet (CD) (providing 28.5% (Kcal) protein, 13.5% fat and 58% carbohydrates) was obtained from Purina LabDiet, Framingham, MA, USA. HFD (providing 20% (Kcal) protein, 60% fat and 20% carbohydrates) was obtained from Research Diets, New Brunswick, NJ, USA. MGF was obtained from CTMedChem (Bronx, NY, USA) and administered orally via food. CD and HFD were ground into fine powder and mixed with pure MGF. The mixtures were re-pelleted and administered to individually housed mice. Mice were fed ad libitum with CD or HFD ± MGF (MGF dose, 400 mg/kg body weight) and water for 16 weeks. Mice were then sacrificed after 12-h fasting from 10 p.m. to 10 a.m. Quadriceps were collected, snap frozen in liquid N_2_ and stored at −80 °C.

### 4.3. Metabolite Profiling

Metabolite profiling was conducted at the Einstein Stable Isotope & Metabolomics Core. There are two main processes. One is sample preparation and the other is LC/MS/MS analysis. Tissue samples were homogenized with a mixture of methanol, acetonitrile and water (methanol:acetonitrile:water = 2:2:1 (*v*:*v*:*v*)) with tissue factor of 6 (to tissue weight). Internal standard (1.8 µg of UC13-succinate) was spiked to each sample before extraction. The samples were homogenized and centrifuged. The supernatant was transferred to a glass tube for lyophilization. The residue was reconstituted in 265 µL of 5 mM ammonium fumarate in acetonitrile solution (acetonitrile:water = 6:4 (*v*:*v*)).

LC/MS/MS (Waters Xevo TQ) analysis was performed to quantitate metabolites using similar method described previously [52]. Chromatographic analysis, prior to targeted and multiple reaction monitoring (MRM) mass spectrometric analyses, was performed using an Aquity UPLC using a Waters BEH amide 1.7 µm column 2.1 × 100 mm, and an acidic mobile phase containing an acetonitrile/water gradient, as per [52]. Samples were bracketed in between calibration standards and linear regression was performed for quantitation.

### 4.4. mRNA Measurement by Real Time qRT-PCR

Total RNA from muscle tissues was extracted with Trizol. One µg of total RNA was used to synthesize cDNA with Reverse Transcriptase and OligodT from Invitrogen (18080051, Carlsbad, CA, USA). Real time PCR using the Sybrgreen master mix (Applied Biosystems, Foster City, CA, USA) was performed by a 7900HT PCR machine from Applied Biosystems. The level of mRNA was calculated versus β-actin transcript and expressed as fold over control. All primers used for real time qRT-PCR are listed in Table 2.

### 4.5. Mitochondria Isolation

Mitochondria were isolated from muscle tissues using previously described method [53,54] with modified buffer (250 mM sucrose, 1 mM EGTA, 10 mM HEPES, and 5 g/L BSA, pH 7.5). Mitochondrial pellets were suspended, and mitochondria were permeabilized by a freeze–thaw procedure before used to evaluate the activity of mitochondrial membrane complexes like complex II (SDH) [53,54].

### 4.6. Measurement of Mitochondrial Complex II Activity

Complex II specific activity was measured by following the decline in absorbance at 600 nm due to the reduction of dichlorophenol indophenols. Two hundred and twenty five microliters of the reaction mixture (containing 50 mM potassium phosphate pH 7.8, 2 mM EDTA, 0.1% BSA, 0.3 mM KCN, 50 mM dichlorophenol indophenol, 3 μM rotenone, 1 μM antimycin A, 2 mmol/L ATP, 50 μM CoQ1 and 45 μg mitochondria protein) was loaded onto 96-well plate and equilibrated at 30 °C for 5 min. The reaction was started by addition of 25 µL sodium succinate at the final concentration of 10 mM and the enzyme-catalyzed reduction of dichlorophenol indophenol was monitored as absorbance at 600 nm for 5 min. Graphs of absorbance versus time were plotted and reaction rates were calculated.

### 4.7. Measurement of ATP Production

ATP production was measured by using the method previously described [55], with some modifications. We reduced the total volume of the reaction mixture from 1 mL to 250 µL and the reaction was carried out in 96-well plates.

### 4.8. Mitochondrial DNA (mtDNA)

Mitochondrial DNA content was determined according to the protocol described by Ajaz et al. [56]. It was quantified by real-time quantitative PCR.

### 4.9. Statistical Analysis

Group measurements were expressed as average ± SEM. One-way ANOVA was used to test for differences among groups. When either a significant (*p* < 0.05) or a marginal (0.05 < *p* < 0.1) effect was observed, two-tailed Student’s *t* tests were conducted to analyze the significance of the differences between two specific groups. *p* < 0.05 was considered significant.

## 5. Conclusions

In this study, we report that MGF significantly increased metabolites in glycolysis, and the increases augmented from the early steps to the late steps, indicating that MGF accelerated glycolytic flux, and this was supported by MGF induced mRNAs of several enzymes catalyzing glycolysis. MGF enhanced overall metabolism in the TCA cycle in a direction from citrate to oxaloacetate. It increased most of metabolites in the TCA cycle, but reduced succinate. MGF enhanced conversion of succinate to fumarate, which occurs not only in the TCA cycle but also in the ETC, by upregulating SDH at both the activity and the expression levels. At the expression level, MGF upregulated not only mtDNA contents, but also mRNAs of most of mitochondrial genes and their transcriptional factors. Overall, MGF upregulated mitochondrial oxidative capacity, which was indicated by MGF-increased ATP production. This study provides molecular understanding of how MGF stimulates carbohydrate oxidation and thereby mitigates hyperglycemia and insulin resistance. As a natural compound, MGF has great potential to be developed into a nutraceutical/pharmaceutical to combat diabetes and obesity.

## Figures and Tables

**Table 1:** Profiles of metabolites in quadriceps of mice.

Metabolite	Chow				HFD				HFD/Chow (Ctl)	
	Ctl	MGF	Ratio (MGF/Ctl)	* *p* (MGF Versus Ctl)	Ctl	MGF	Ratio (MGF/Ctl)	* *p* (MGF Versus Ctl)	Ratio	* *p*
UDPG	7.95	7.63	0.96	0.115	6.40	5.89	0.92	0.316	0.80	0.098
Glucose-6-phosphate	6.93	8.90	1.28	0.272	13.19	18.84	1.43	0.018	1.90	0.034
Fructose-1,6-bisphosphate	44.17	58.90	1.33	0.099	54.28	76.40	1.41	0.043	1.23	0.451
3-Phosphoglycerate	26.03	34.09	1.31	0.089	28.39	46.18	1.63	0.030	1.09	0.445
Phosphoeolpyruate	1.58	2.18	1.38	0.054	0.89	2.27	2.54	0.024	0.57	0.025
Pyruvate	20.48	25.11	1.23	0.175	15.41	18.94	1.23	0.083	0.75	0.038
Lactate	350.59	272.19	0.78	0.046	313.57	213.15	0.68	0.029	0.89	0.144
Glycerol-3-phosphate	120.48	131.00	1.09	0.329	103.34	133.68	1.29	0.152	0.86	0.274
Ribose-5-phosphate	0.39	0.43	1.09	0.216	0.44	0.47	1.05	0.688	1.13	0.406
6-Phosphogluconate	1.40	1.58	1.13	0.193	1.19	1.07	0.89	0.217	0.85	0.130
α-Ketoglutarate	0.45	0.67	1.48	0.043	0.28	0.62	2.20	0.041	0.63	0.035
Succinate	50.81	31.23	0.61	0.038	36.96	21.85	0.59	0.032	0.73	0.050
Fumarate	8.17	11.24	1.38	0.049	6.01	9.09	1.51	0.041	0.74	0.049
Malate	43.89	48.31	1.10	0.121	35.64	40.45	1.13	0.322	0.81	0.084
Aspartate	26.48	29.53	1.12	0.090	19.45	20.82	1.07	0.210	0.73	0.042

C57BL6/J mice were fed chow or HFD and treated with MGF or without MGF for 16 weeks. Mice were then sacrificed after fasting for 12 h from 10 p.m. to 10 a.m. Their quadriceps were collected and snap frozen in liquid N_2_. Metabolites were analyzed by the metabolomics study. Values are average, *n* = 6. * *p* < 0.05 is considered significant. HFD, high fat diet; MGF, mangiferin; Ctl, control; UDPG, urindine diphosphate glucose.

**Table 2:** Mouse primers for real time qRTPCR.

Gene	Forward Sequence from 5′ to 3′	Reverse Sequencefrom 5′ to 3′
*HK2*	TGATCGCCTGCTTATTCACGG	AACCGCCTAGAAATCTCCAGA
*PGI*	TGGCCCAGACTGAGGCCCTG	CTCTAGGTGTCTTTATTCTA
*PFK*	CAGATCAGTGCCAACATAACCAA	CGGGATGCAGAGCTCATCA
*FBA*	AGCCTTCTGAGAAGGATGCTC	GTCCAGCATGAAGCAGTTGAC
*GAPDH*	ACTGGCATGGCCTTCCG	CAGGCGGCACGTCAGATC
*PGK1*	GGTCTTGCCAAAATGTCGCT	TCCAATCGAATCCTCGTGTC
*PGMb*	TGCCCGTCGCCATGGCTGCC-	CGGTGGGACATCATAAGATC-
*Enolase1*	GCCGGCTTTACGTTCACCTC	GTTGAAGCACCACTGGGCAC
*CS*	AAGGACGAGGCAGGATGAG	TGCAGCTGTAGCTCTCTCCC
*Idh1*	ATTGGTGGCATCACGATTCT	TGGAGATGCAAGGAGATGAA
*AKGDH*	AGTGGTGGTGGGTAAGTGGA	GCAAAACTTGATCCTCTCGG
*Suclg1*	TGTTTCCGAGAGGCTGTGTA	CAACCATGGTCTCCAGCAG
*SDHb*	GTCTGTGCCCCTCGACAG	TGACGTCAGGAGCCAAAAT
*FH1*	ACACGGAAGGAATTTTGGCT	ACCATGTACCGCGCACTC
*MDH1*	TGCTCCAGTCACAAGGACTC	GACTGCTGGAGACTGCCTTT
*CoxI (Ndufa9)*	TAAGGGATGAAGGTCCGATG	GATCCAGATGCCGTAGGAAA
*Cyc (cytochrome c)*	GGAGGCAAGCATAAGACTGG	TCCATCAGGGTATCCTCTCC
*CoxIV*	AGAAGGAAATGGCTGCAGAA	GCTCGGCTTCCAGTATTGAG
*Atp5g1*	GCTGCTTGAGAGATGGGTTC	AGTTGGTGTGGCTGGATCA
*PGC-1α*	AAGTGTGGAACTCTCTGGAACTG	GGGTTATCTTGGTTGGCTTTATG
*PGC-1β*	TGCTGCTGTCCTCAAATACG	TGGAGACTGCTCTGGAAGGT
*NRF*	CGGAGTGACCCAAACTGAAC	GATGACCACCTCGACCGTTT
*ERRα*	ACTGCCACTGCAGGATGAG	CACAGCCTCAGCATCTTCAA
*TFAM*	CCAAAAAGACCTCGTTCAGC	ATGTCTCCGGATCGTTTCAC
*Β-actin*	CATGGAGTCCTGTGGCATC	AGCACTGTGTTGGCGTAC

## Abbreviations

TCA	Tricarboxylic acid
ETC	Electron transport chain
PDH	Pyruvate dehydrogenase
CS	Citrate synthase
α-KG	α-Ketoglutarate
α-KGDH	α-Ketoglutarate dehydrogenase
PFK	Phosphofructose kinase
GAPDH	Glyceraldehyde 3-phosphate dehydrogenase
HFD	High fat diet
MGF	Mangiferin
UDPG	Urindine diphosphate glucose
PEP	Phosphoenolpyruvate
HK	Hexose kinase
PGI	Phosphoglucose isomerase
PFK	Phosphofructokinase
FBA	Fructose-1,6-bisphosphate aldolase
PGK	Phosphoglycerate Kinase
PGM	Phosphoglycerate mutase
PK	Pyruvate kinase
LDH	Lactate dehydrogenase
PC	Pyruvate carboxylase
CS	Citrate synthase
AC	Aconitase
IDH	Isocitrate dehydrogenase
Sucl	Succinyl coenzyme A synthetase
SDH	Succinate dehydrogenase
FH	Fumarase
MDH	Malate dehydrogenase
Atp5g1	ATP synthase subunit 5g1
TFAM	Transcriptional factor A
NRF1	Nuclear respiratory factor 1
ERRα	Estrogen-related receptor α
PGC-1α	Peroxisome proliferator-activated receptor-γ coactivator-1α
PGC-1β	Peroxisome proliferator-activated receptor-γ coactivator-1β
mtDNA	Mitochondrial DNA
PBS	Phosphate-buffered saline
